# Relationship Between Sleep and Meal Timing with Glycemia Parameters in Individuals with Obesity Participating in a Randomized Time-Restricted Eating Study

**DOI:** 10.3390/nu18111824

**Published:** 2026-06-05

**Authors:** Sirimon Reutrakul, Stacey L. Simon, Qi Wang, Emily N. C. Manoogian, Satchidananda Panda, Suryeon Ryu, Zan Gao, Caleb Griffiths, Erika Helgeson, Douglas G. Mashek, Niki Oldenburg, Lisa Senye Chow

**Affiliations:** 1 Department of Medicine, University of Illinois Chicago, Chicago, IL 60612, USA; sreutrak@uic.edu; 2 Department of Pediatrics, University of Colorado Anschutz Medical Campus & Children’s Hospital, Aurora, CO 80045, USA; stacey.simon@childrenscolorado.org; 3 Clinical and Translational Science Institute, University of Minnesota, Minneapolis, MN 55414, USA; wangx890@umn.edu; 4 Regulatory Biology Laboratory, Salk Institute for Biological Sciences, La Jolla, CA 92037, USA; emanoogian@salk.edu (E.N.C.M.); panda@salk.edu (S.P.); 5 Center for Children’s Healthy Lifestyles & Nutrition, Children’s Mercy Kansas City, Kansas City, MO 64108, USA; sryu@cmh.edu; 6 Kinesiology, Recreation, and Sport Studies, College of Education, Health, and Human Sciences, University of Tennessee, Knoxville, TN 37996, USA; zan@utk.edu; 7 School of Public Health, University of Minnesota, Minneapolis, MN 55455, USA; grif0461@umn.edu (C.G.); helge@umn.edu (E.H.); 8 Department of Biochemistry, Molecular Biology and Biophysics, Medical School, Masonic Institute on the Biology of Aging, University of Minnesota, Minneapolis, MN 55455, USA; dmashek@umn.edu; 9 Department of Medicine, Medical School, University of Minnesota, Minneapolis, MN 55455, USA; olden019@umn.edu

**Keywords:** circadian rhythms, eating window, chrononutrition, glycemic control, continuous glucose monitoring, metabolic health

## Abstract

**Background/Objectives:** Circadian misalignment, including mistimed sleep or eating, is associated with altered glucose metabolism. The importance of eating window timing for time-restricted eating (TRE) is increasingly recognized. This secondary analysis examined associations between meal to sleep timing intervals and glycemic parameters in individuals with obesity across three dietary interventions [TRE, CR: caloric restriction, and UE: unrestricted eating]. **Methods:** Participants aged 18–65 years with obesity were randomized to a 12-week intervention: TRE (8 h eating window), CR (15% reduction in daily caloric intake), or UE (usual eating habits). CGM and actigraphy were assessed over two weeks at baseline and end-intervention. Mixed effects models examined associations between continuous glucose monitoring (CGM) outcomes and two intervals: last meal to sleep onset (PM meal-Sleep) and awakening to first meal (Awake-AM meal). **Results:** Each hour increase in the Awake-AM meal interval was associated with lower overnight (1 AM–5 AM) average glucose, lower glycemic variability, lower %time > 180 mg/dL, and greater %time < 70 mg/dL. Each hour increase in the PM meal-Sleep interval was associated with lower overnight (1 AM–5 AM) average glucose. Both associations persisted after adjustment for baseline sleep duration, HbA1c, and randomization assignment. **Conclusions:** In individuals with obesity, morning (Awake-AM meal interval) and evening (PM meal-Sleep interval) fasting relative to sleep were differentially associated with glycemic control. These findings highlight the relevance of eating and sleep timing to glycemic parameters and may inform eating window selection for individuals practicing TRE.

## 1. Introduction

The circadian system, governed by the master clock in the hypothalamic suprachiasmatic nuclei (SCN), regulates daily rhythms including the sleep–wake cycle, feeding behavior, and central and peripheral energy metabolism [1]. The primary zeitgeber (time-giver) for this system is light, perceived by the retina and relayed to the SCN via the retinohypothalamic tract. Through hormonal and neuronal pathways, this central clock synchronizes peripheral clocks throughout the body to ensure coordinated physiological rhythms [1]. Beyond light, meal timing serves as a major zeitgeber for peripheral clocks, exerting significant influence on systemic energy metabolism.

Circadian misalignment, resulting from mistimed sleep, light exposure, or eating, drives adverse health outcomes including impaired glucose metabolism, systemic inflammation, obesity, and increased cardiovascular mortality [2,3,4]. Night-shift workers, who eat and sleep out of sync with natural circadian rhythms, face significantly higher risks of hypertension, diabetes, and cancer, as well as poorer glycemic control [5]. Even subtle circadian shifts, such as delayed sleep or meal timing, are linked to metabolic dysfunction. For instance, individuals with a late chronotype have an increased risk of diabetes and poorer glycemic control [6]. Population-based data further show that every one-hour delay in the start of daily eating is associated with a 0.6% increase in fasting glucose and a 3% increase in insulin resistance [7]. Similarly, higher late-day caloric intake and later timing of the final meal are linked to abdominal obesity, elevated fasting glucose, and weight-loss resistance [8,9]. Critically, clock time may differ from internal circadian timing. Research in young adults demonstrates that food intake relative to melatonin onset, a primary biological marker, is significantly associated with body fat and BMI, whereas clock time alone is not [10].

Time-restricted eating (TRE) is an increasingly popular dietary strategy that limits food intake to a 4–12 h daily window, typically 8–10 h, allowing ad libitum consumption during that period [11]. While initial TRE studies focused primarily on window duration, recent studies have begun comparing early TRE (window starting before 10 am, ending at or before 6 pm) versus late TRE (window starting at noon), reflecting growing recognition of circadian timing. [12]. A recent meta-analysis of 41 randomized studies found that early TRE significantly reduced body weight (mean difference: 1.15 kg) and fasting insulin concentrations compared to late TRE [13]. Although these studies categorized TRE by clock time, the practice inherently shifts meal timing relative to other circadian-regulated behaviors, such as sleep. Therefore, we examined the relationship between sleep–meal timing gaps and continuous glucose monitoring (CGM) outcomes to evaluate the association of TRE with glucose metabolism, leveraging CGM to capture high-resolution metrics including mean glucose and glucose distribution over 24 h and overnight periods [14]. This secondary analysis explored the relationship between meal to sleep intervals and glycemic parameters in a cohort with obesity participating in a randomized trial comparing TRE, caloric restriction (CR), and unrestricted eating (UE). We focused on two intervals: (1) awakening to first meal (Awake-AM meal), and (2) last meal to sleep onset (PM meal-Sleep). We hypothesized that greater temporal separation between meals and sleep, both in the morning (Awake-AM meal interval) and evening (PM meal-Sleep interval), would improve CGM-derived glycemic measures over 24 h and during the overnight period (1–5 AM).

## 2. Materials and Methods

### 2.1. Design Overview

This is a secondary analysis of a previously published trial [15]. This 12-week randomized controlled trial (2020–2023, ClinicalTrials.gov NCT04259632, registration date 04 February 2020) randomized participants 1:1:1 to TRE (8 h eating window), CR (15% caloric reduction), or usual eating (UE) control. The primary study design and participant flow have been published previously [15]. In brief, computer-generated randomization in Research Electronic Data Capture (REDCap) [16] used permuted blocks of three, stratified by sex and age (<45 vs. ≥45 years), to assign participants to the TRE, CR, or UE groups. The statistical team generated and stored the randomization sequence in REDCap before study initiation. Participants were unblinded, whereas outcome assessors remained blinded. The study received Institutional Review Board (IRB) approval from the University of Minnesota (UMN: STUDY00008545) and Salk Institute (15-0003), and all participants signed the consent form prior to enrollment. Participants documented all eating occasions using the mCC app [15]. CONSORT diagram for the primary study is in Supplemental Figure S1.

### 2.2. Setting and Participants

As previously described [15], participants aged 18–65 years with Body Mass Index (BMI) 30–55 kg/m^2^ were recruited from MHealth Fairview Health System in Minneapolis, MN. Inclusion criteria required self-reported wake times of 5–9 AM, 6–9 h of sleep per night, stable weight (±5 pounds for >3 months), English proficiency, and a smartphone compatible with a mobile phone app [myCircadianClock: mCC: https://www.mycircadianclock.org, accessed on 2 June 2026], a digital food tracker that captures eating occasions via photo, text description, and time/date stamp [17]. Participants needed ≥7 days of mCC documentation showing ≥2 daily eating events separated by ≥5 h, recorded >1 day/week, with a ≥12 h eating window (95% of events). Exclusion criteria included eating window <12 h, weight-affecting medications, diabetes, shift work, major medical conditions, MRI contraindications, pregnancy or planned pregnancy, illiteracy, and eating disorders [18].

### 2.3. Measures

#### 2.3.1. mCC App Documentation of Dietary Intake

All participants, regardless of group, used the mCC app [17] to record all oral intake throughout the study, except water and medications. An eating occasion was defined as any instance of oral intake, including coffee, tea, diet drinks, and noncaloric beverages. The daily eating time was noted as the first and last logged eating time as captured on the mCC app. For this secondary analysis, mCC app data were collected concurrently with CGM assessments for 10–14 days at baseline (before randomization) and at end-intervention.

#### 2.3.2. CGM Assessment

The CGM (blinded Dexcom G6 Pro, Dexcom, San Diego, CA, USA) was worn for ~10–14 days prior to randomization and for 2 weeks prior to end-intervention. Calculated metrics included standard CGM measures: average glucose (mg/dL), SD, CV, %time > 180 mg/dL, %time < 70 mg/dL, and time-in-range (TIR:70–180 mg/dL). Additional metrics were calculated using the R package rGV [14] and included area under the CGM curve (AUC-CGM), low blood glucose index (LBGI) [19], and high blood glucose index (HBGI) [20], which highlight glycemic extremes. CGM metrics were calculated across each day during the participant’s 10-day wear period and, additionally, across each overnight window (1 AM–5 AM).

#### 2.3.3. Objective Sleep Assessment

Participants wore ActiGraph GT9X Link accelerometers (ActiGraph LLC, Pensacola, FL, USA) on the non-dominant wrist for two weeks at baseline and end-intervention. Devices recorded raw triaxial acceleration at 30 Hz, processed by GGIR into 5 s epochs. Sleep onset and wake time during each recording period were estimated from raw accelerometer data using the GGIR R package (v2.9.1) [21]. GGIR identified sustained inactivity bouts using an angle-based method (*z*-axis angle variability <5 degrees sustained for ≥5 min) and detected sleep period time windows using the HDCZA algorithm, which does not require concurrent sleep logs [22]. Nights were considered valid if ≥16 h of valid data were available within the 24 h window used for sleep scoring.

### 2.4. Statistical Analysis

The analysis was restricted to participants with sleep, mCC, and CGM data at both baseline and 12-week timepoints. Participant baseline characteristics were summarized using descriptive statistics. Groups were compared using ANOVA for variables presented as mean (SD) and Kruskal–Wallis test for variables presented as median (IQR). Categorical variables were compared between groups using Fisher’s exact test. Sleep timing, eating occasions, and CGM variables were assessed for every day of available recorded data at end-intervention. Mixed effects models using subject specific random intercepts were used on the daily data to assess associations between CGM outcomes and two meal timing intervals: last to sleep onset (PM meal-Sleep interval) and awakening to first meal (Awake-AM meal interval). Analyses were performed at end-intervention, as the greater variability in timing intervals resulting from TRE provided insight into these relationships. Primary models were adjusted for randomization assignment only. Secondary models additionally adjusted for baseline sleep duration, HbA1c, and randomization assignment. The relationship between Awake-AM meal interval and the PM meal-Sleep interval was assessed using a mixed effects model with subject specific random intercepts adjusting for randomization assignment. Paired t-tests were used to compare changes in CGM variables between baseline and end-intervention.

Analyses were performed in SAS version 9.4 (SAS Institute Inc., Cary, NC, USA). *p* values of less than 0.05 were considered statistically significant. No adjustment for multiple testing was made.

## 3. Results

Characteristics of participants (n = 44) are shown in Table 1. Mean (SD) age was 42.8 (11.5) years with a BMI of 36.6 (5.3) kg/m^2^; the majority were female. Meal start and end times at baseline and end-intervention, average wake time and bedtime, and baseline glycemic parameters are also shown in Table 1. On average, each participant contributed 9.3 (2.1) days of observations. At end-intervention, participants tended to reduce their eating interval by ending their eating period earlier: median eating time started 33 min later and ended 68 min earlier, with most changes occurring in the TRE group compared to CR and UE. Wake time and bedtime did not change significantly during the study.

Supplemental Table S1 shows changes in CGM variables between baseline and end-intervention, with a significant reduction in overnight average glucose (*p* = 0.043).

**Figure 1:**
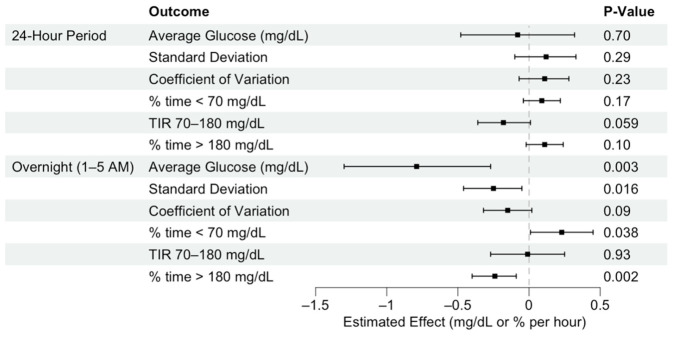
At end-intervention, effect of a one-hour increase in Awake-AM meal interval on CGM measures, adjusting for randomization assignment.

We found that each additional hour increase in the Awake-AM meal interval was associated with 0.19 (95% CI: 0.10 to 0.28) fewer hours for the PM meal-Sleep interval, suggesting that a later first meal compresses the time between the last meal and sleep onset. In Figure 1**:** each one-hour increase in the Awake-AM meal interval was associated with lower overnight (1–5 AM) average glucose and glycemic variability (SD), reduced %time > 180 mg/dL, and increased %time < 70 mg/dL.

Fully adjusted models (Table 2; covariates: baseline sleep duration, HbA1c, and randomization assignment) confirm these associations.

**Figure 2:**
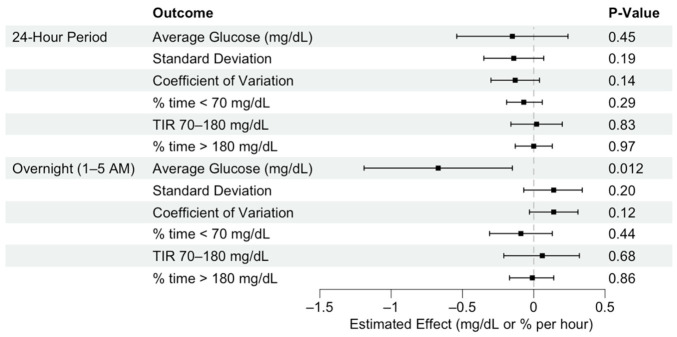
At end-intervention, effect of a one-hour increase in the PM meal-Sleep interval on CGM measures, adjusting for randomization assignment.

In Figure 2, each one-hour increase in the PM meal-Sleep interval was associated with lower overnight (1–5 AM) average glucose, and this association persisted after adjustment for the same covariates (Table 3).

## 4. Discussion

In this secondary analysis of participants with obesity enrolled in a randomized trial of dietary interventions (TRE vs. CR vs. UE), each hour increase in the Awake-AM meal interval was associated with lower overnight (1 AM–5 AM) average glucose, lower glucose variability (SD), increased time below target, and decreased time above target. Each hour increase in the PM meal-Sleep interval was associated with lower overnight average glucose. Both associations persisted after adjustment for baseline sleep duration, HbA1c, and randomization assignment. These findings demonstrate that morning and evening meal timing, relative to wake time and bedtime, respectively, were differentially associated with 24 h and overnight CGM measures, with relevance to eating window selection for individuals practicing TRE.

In the primary paper, we found no differences in CGM-measured mean glucose or TIR between 3 months of TRE, CR (15% reduction), and UE among patients without diabetes [15]. These results contrast with a 6-month trial in patients with type 2 diabetes, where TRE reduced mean glucose compared to unrestricted eating [23]; however, that difference was primarily driven by rising glucose in the UE group, as the TRE group showed no significant improvement from baseline.

Increasing the Awake-AM meal interval was associated with reduced overnight glucose, glycemic variability (SD), and time above target (>180 mg/dL), while increasing time below target (<70 mg/dL); these findings warrant discussion. Although prolonged AM fasting was associated with a slight increase in time <70 mg/dL, this remained <1% in participants without diabetes, consistent with healthy ranges [24]. One potential explanation for these findings is that prolonged morning fasting disrupts circadian rhythm and blunts AM cortisol [25], an effect also observed in Ramadan [26,27]. Since meal timing influences peripheral clocks, prolonged morning fasting may speculatively influence overnight glycemia through circadian entrainment, thereby reducing hepatic glucose output. Despite these glucose-lowering findings, ample evidence supports the benefit of early eating, which generally corresponds to shorter Awake-AM meal intervals. Early TRE appears more effective than late TRE for improving fasting glucose, body weight, and blood pressure [13,28]. Chronic breakfast skipping is associated with increased risks of obesity, diabetes, and poor glycemic control [29,30], although the reduced caloric intake with TRE [15] may offset the adverse glycemic effects of a shorter PM meal-sleep interval, a known consequence of breakfast skipping [31]. Caution is warranted for individuals using insulin or insulin secretagogues during prolonged overnight fasts due to the potential risk of hypoglycemia, consistent with American Diabetes Association recommendations [32].

Our results (unadjusted and adjusted) suggest that a longer PM meal-Sleep interval reduces overnight average glucose. These findings contribute to the growing literature on the adverse effects of late eating on cardiometabolic parameters. For instance, National Health and Nutrition Examination Survey (NHANES) data linked late-day eating to a 12% higher risk of abdominal obesity and a 65% increased risk of elevated fasting glucose [8]. Furthermore, late eaters in weight-loss programs face greater biological barriers and lower motivation than early eaters [33], while night eating in patients with diabetes correlates with poor glycemic control [34]. Mechanistically, late eating impairs pre-meal carbohydrate utilization, increases postprandial and overnight glucose, blunts cortisol profiles [35], and reduces overnight fatty acid oxidation [36]. Consuming carbohydrates during peak endogenous melatonin levels further impairs glucose tolerance, particularly in carriers of the MTNR1B risk allele [31,37].

This study’s strengths include day-to-day analysis of objective sleep assessments, documentation of eating occasions, and CGM parameters assessed at end-intervention, which may capture greater variability in eating–sleeping intervals than baseline. Limitations include the exploratory nature of this analysis, small sample size, and use of pooled samples, although we adjusted for randomization assignment. We also lacked a gold-standard circadian timing measure, such as dim light melatonin onset, which could further characterize the relationship between meal timing and internal circadian markers. Our participants did not have diabetes, an important consideration when interpreting our findings, and may explain the modest effect sizes on glucose parameters, as glycemic variability is generally lower in this population than in individuals with diabetes. Finally, as an exploratory analysis, we did not adjust for multiple comparisons.

## 5. Conclusions

In conclusion, a longer Awake-AM meal interval was associated with lower overnight average glucose, less glycemic variability, and less hyperglycemia, but at the cost of increased hypoglycemia risk. A longer PM meal-Sleep interval was associated with lower overnight average glucose without increasing hypoglycemia risk. These findings suggest that delaying breakfast may be associated with improved CGM-assessed glycemic measures more than an earlier dinner, albeit with greater hypoglycemia risk, making a longer PM meal-Sleep interval preferable for individuals at risk for hypoglycemia. Taken together, meal timing relative to sleep is associated with alterations in CGM metrics, highlighting the potential to personalize TRE programs based on individual glycemic risk profiles.

## Figures and Tables

**Table 1:** Demographic characteristics, eating and sleeping timing at baseline and end-intervention, and baseline glucose parameters of the participants. * The first meal time (2.5% eating time) was defined as the point at which <2.5% of eating occasions had occurred; the last meal time (97.5% eating time) was defined as the point at which ≥97.5% of occasions had occurred over the 2-week observation period [15].

	Overall (N = 44)	TRE (N = 19)	CR (N = 8)	UE (N = 17)	*p* Value
**Demographics**					
Age (years), mean (SD)	42.8 (11.5)	43.6 (12.4)	40.4 (12.2)	43.2 (10.8)	0.80
Gender, n (%)					0.15
Female	26 (59.1%)	8 (42.1%)	6 (75.0%)	12 (70.6%)	
Male	18 (40.9%)	11 (57.9%)	2 (25.0%)	5 (29.4%)	
Race, n (%)					1
Black or African American	2 (4.5%)	1 (5.3%)	0 (0%)	1 (5.9%)	
Mixed	1 (2.3%)	1 (5.3%)	0 (0%)	0 (0%)	
White	41 (93.2%)	17 (89.5%)	8 (100.0%)	16 (94.1%)	
Weight (kg), mean (SD)	109.7 (20.1)	114.1 (22.6)	99.8 (15.0)	109.4 (18.4)	0.24
BMI (kg/m^2^), mean (SD)	36.6 (5.3)	36.7 (6.4)	35.1 (4.7)	37.3 (4.4)	0.65
**Average number of days with available samples for analysis at end-intervention, mean (SD)**	9.3 (2.1)	9.1 (2.5)	9.0 (2.1)	9.7 (1.5)	0.69
**Timings—Baseline, median (IQR)**					
Eating window duration (hours): mean (SD)	14.3 (1.0)	14.3 (0.8)	14.1 (1.3)	14.5 (1.0)	0.70
Eating window start (2.5) (hh:mm) *	7:20 (6:41–8:05)	7:32 (6:43–8:05)	7:40 (6:53–8:34)	7:07 (6:32–7:38)	0.40
Eating window end (97.5) (hh:mm) *	21:29 (21:01–22:25)	21:29 (20:47–22:26)	21:37 (21:13–22:11)	21:29 (21:08–22:00)	0.94
Sleep duration (hours): mean (SD)	6.8 (0.8)	7.0 (0.6)	6.8 (0.8)	6.6 (0.9)	0.27
Wake time (hh:mm)	6:53 (6:10–7:37)	6:59 (6:09–7:30)	7:21 (6:45–8:04)	6:28 (6:06–7:00)	0.17
Bedtime (hh:mm)	23:02 (22:27–23:50)	22:47 (21:53–23:47)	23:32 (22:58–24:22)	22:52 (22:28–23:55)	0.46
Awake-AM meal interval (hh:mm)	0:28 (0:00–0:58)	0:29 (0:00–0:49)	0:10 (0:00–1:03)	0:33 (0:11–0:58)	0.81
PM meal-Sleep interval (hh:mm)	1:29 (0:52–2:02)	1:20 (0:39–1:38)	1:52 (1:10–2:22)	1:23 (1:01–1:57)	0.28
**Timings—End-intervention, median (IQR)**					
Eating window duration (hours): mean (SD)	11.7 (2.8)	9.1 (1.7)	13.4 (1.3)	13.8 (1.6)	<0.0001
Eating window start (2.5) (hh:mm) *	7:52 (7:08–10:14)	10:30 (8:02–11:02)	7:23 (6:33–7:59)	7:18 (6:39–7:48)	<0.0001
Eating window end (97.5) (hh:mm) *	19:59 (18:54–20:52)	19:00 (18:30–19:49)	20:18 (19:59–22:03)	20:59 (20:17–22:05)	<0.0001
Sleep duration (hours): mean (SD)	6.8 (0.8)	7.0 (0.6)	6.8 (0.8)	6.6 (0.9)	0.23
Wake time (hh:mm)	6:53 (5:36–7:26)	6:48 (6:01–7:19)	7:06 (5:42–7:55)	6:46 (5:24–7:11)	0.71
Bedtime (hh:mm)	22:58 (22:05–23:41)	23:04 (22:06–23:40)	22:55 (21:32–25:01)	22:58 (22:13–23:30)	0.95
Awake-AM meal interval (hh:mm)	1:16 (0:30–3:25)	3:52 (1:36–4:45)	0:38 (0:13–0:58)	0:54 (0:04–1:24)	0.0003
PM meal-Sleep interval (hh:mm)	2:49 (1:35–3:56)	3:54 (3:06–4:38)	1:48 (1:30–3:16)	1:39 (0:57–2:31)	0.0008
**Change in eating and sleeping time before and after intervention, median (IQR)**					
Change in eating window hours: mean (SD)	−2.6 (2.8)	−5.2 (2.1)	−0.8 (1.1)	−0.7 (1.3)	<0.0001
Change in eating window start (hh:mm)	0:33 (−0:02 to 2:08)	2:47 (0:59 to 4:27)	−0:10 (−0:37 to 0:22)	0:11 (−0:02 to 0:28)	<0.0001
Change in eating window end (hh:mm)	−1:08 (−2:39 to −0:19)	−2:26 (−3:39 to −1:25)	−1:03 (−1:59 to −0:13)	−0:29 (−0:37 to 0:19)	0.0009
Change in wake time (hh:mm)	−0:09 (−0:41 to 0:09)	−0:01 (−0:24 to 0:09)	−0:23 (−0:48 to −0:15)	−0:07 (−0:46- to 0:35)	0.27
Change in bedtime (hh:mm)	−0:07 (−0:40 to 0:32)	−0:04 (−0:31 to 0:38)	−0:20 (−1:26 to 0:21)	−0:14 (−0:41 to 0:19)	0.44
Change in Awake-AM meal interval (hh:mm)	0:44 (0:00 to 2:34)	2:47 (0:53 to 4:27)	0:03 (−0:11 to 0:25)	0:13 (−0:06 to 0:58)	<0.0001
Change in PM meal-Sleep interval (hh:mm)	1:01 (−0:02 to 2:38)	2:38 (1:26 to 3:23)	0:43 (0:06 to 0:56)	0:00 (−0:21 to 0:46)	0.0003
**Glycemic Measures at baseline, mean (SD)**					
Hemoglobin A1C (%)	5.5 (0.4)	5.5 (0.4)	5.2 (0.3)	5.6 (0.3)	0.08
Homeostatic Model Assessment of Insulin Resistance (HOMA-IR)	4.2 (2.9)	3.8 (2.3)	3.2 (2.6)	5.2 (3.6)	0.20
Fasting glucose (mg/dL)	95.3 (7.8)	95.8 (7.7)	91.3 (6.1)	96.6 (8.4)	0.27
Fasting insulin (mU/L)	18.3 (12.0)	16.2 (10.4)	16.1 (10.5)	21.6 (14.1)	0.37

Groups were compared using ANOVA for variables presented as mean (SD), Kruskal–Wallis test for variables presented as median (IQR), and Fisher’s exact test for gender and race.

**Table 2:** At end-intervention, effect of a one-hour increase in Awake-AM meal interval on CGM measures. Results are from mixed effects models adjusting for baseline sleep duration, HbA1c, and randomization assignment.

Outcome	Estimated Effect (95% CI) for Every 1 h Increase in the Awake-AM Interval	*p* Value
**CGM measures over 24 h**		
Average glucose (mg/dL)	−0.08 (−0.48, 0.32)	0.71
Standard Deviation	0.12 (−0.09, 0.34)	0.27
Coefficient of Variation	0.11 (−0.06, 0.29)	0.21
% time spent in glucose below target (<70 mg/dL)	0.10 (−0.03, 0.23)	0.14
% time spent in target glucose (70–180 mg/dL)	−0.18 (−0.36, 0)	0.05
% time spent in glucose above target (>180 mg/dL)	0.11 (−0.02, 0.24)	0.10
**CGM measures during overnight period (1 AM–5 AM)**		
Average glucose (mg/dL)	−0.77 (−1.29, −0.25)	0.004
Standard Deviation	−0.25 (−0.46, −0.04)	0.017
Coefficient of Variation	−0.15 (−0.32, 0.02)	0.08
% time spent in glucose below target (<70 mg/dL)	0.24 (0.03, 0.46)	0.029
% time spent in target glucose (70–180 mg/dL)	−0.04 (−0.30, 0.22)	0.77
% time spent in glucose above target (>180 mg/dL)	−0.22 (−0.37, −0.07)	0.003

**Table 3:** At end-intervention, effect of a one-hour increase in the PM meal-Sleep interval on CGM measures. Results are from mixed effects models adjusting for baseline sleep duration, HbA1c, and randomization assignment.

Outcome	Estimated Effect (95% CI) for Every 1 h Increase in the PM Meal-Sleep Interval	*p* Value
**CGM measures over 24 h**		
Average glucose (mg/dL)	−0.16 (−0.55, 0.23)	0.42
Standard Deviation	−0.15 (−0.36, 0.06)	0.16
Coefficient of Variation	−0.13 (−0.30, 0.04)	0.12
% time spent in glucose below target (<70 mg/dL)	−0.07 (−0.20, 0.05)	0.26
% time spent in target glucose (70–180 mg/dL)	0.03 (−0.14, 0.21)	0.70
% time spent in glucose above target (>180 mg/dL)	0 (−0.13, 0.13)	0.97
**CGM measures during overnight period (1 AM–5 AM)**		
Average glucose (mg/dL)	−0.69 (−1.21, −0.17)	0.01
Standard Deviation	0.13 (−0.08, 0.34)	0.21
Coefficient of Variation	0.14 (−0.03, 0.32)	0.11
% time spent in glucose below target (<70 mg/dL)	−0.10 (−0.33, 0.12)	0.37
% time spent in target glucose (70–180 mg/dL)	0.11 (−0.16, 0.38)	0.44
% time spent in glucose above target (>180 mg/dL)	−0.02 (−0.17, 0.13)	0.79

## Data Availability

Protocol: posted as data supplement on website. Documented analytic dataset: Data available 1-year post-publication with IRB approval and MTAs, upon request to Dr. Chow’s team. This data will be provided de-identified. Statistical Code: Code available with dataset upon request to facilitate interpretation.

## Supplementary Materials

The following supporting information can be downloaded at: https://www.mdpi.com/article/10.3390/nu18111824/s1, Table S1: CGM measures at baseline and end-intervention; Figure S1: Consort for primary study published previously.

## Abbreviations

The following abbreviations are used in this manuscript:

TRE	Time-restricted eating
CR	Caloric restriction
UE	Unrestricted eating
