# Supplementary material for: Relationship Between Sleep and Meal Timing with Glycemia Parameters in Individuals with Obesity Participating in a Randomized Time-Restricted Eating Study
Source: Nutrients. 2026 Jun 5;18(11):1824. doi: 10.3390/nu18111824 (PMC13259180; doi:10.3390/nu18111824)
Supplement: Supplementary file 1 [file nutrients-18-01824-s001.zip › nutrients-4293982-supplementary.pdf]

Table S1: CGM measures at baseline and end-intervention**.**

	**Baseline (N=44)**	**End-intervention (N=44)**	**Difference between baseline and end-intervention (N=44)**	**P value**
**CGM measures over 24 hours [median, IQR)]**				
Average glucose (mg/dL)	118.3 (115.3,121.2)	117.2 (113.6,120.8)	-1.1 (-4.3,2.2)	0.52
Standard Deviation	19.5 (18.2,20.7)	19.5 (17.9,21.0)	0 (-1.1,1.2)	0.98
Coefficient of Variation	16.5 (15.5,17.4)	16.6 (15.4,17.7)	0.1 (-0.8,1.1)	0.83
% time spent in glucose below target (<70 mg/dL)	0.6 (0.3,0.9)	0.8 (0.4,1.2)	0.1 (-0.3,0.6)	0.54
% time spent in target glucose (70-180 mg/dL)	98.2 (97.8,98.7)	97.6 (96.9,98.4)	-0.6 (-1.3,0.1)	0.10
% time spent in glucose above target (>180 mg/dL)	1.2 (0.8,1.6)	1.6 (0.9,2.3)	0.4 (-0.1,1.0)	0.14
**CGM measures during overnight period (1 AM–5 AM) [median, IQR)]**				
Average glucose (mg/dL)	120.0 (116.3,123.8)	116.4 (112.5,120.4)	-3.6 (-7.1, -0.1)	0.043
Standard Deviation	12.7 (11.0,14.5)	12.7 (11.1,14.2)	-0.1 (-1.7,1.6)	0.95
Coefficient of Variation	10.5 (9.2,11.7)	10.9 (9.6,12.2)	0.4 (-0.8,1.7)	0.48
% time spent in glucose below target (<70 mg/dL)	0.3 (0.1,0.5)	0.8 (0.2,1.4)	0.5 (-0.1,1.2)	0.09
% time spent in target glucose (70-180 mg/dL)	99.0 (98.2,99.8)	98.8 (98.0,99.5)	-0.2 (-1.4,0.9)	0.67
% time spent in glucose above target (>180 mg/dL)	0.7 (-0.1,1.5)	0.4 (-0.1,0.9)	-0.3 (-1.2,0.6)	0.52

Data presented are median, IQR, P values are from paired t test.

Figure S1: Consort for primary study published previously (Oldenburg, Obesity 2025)

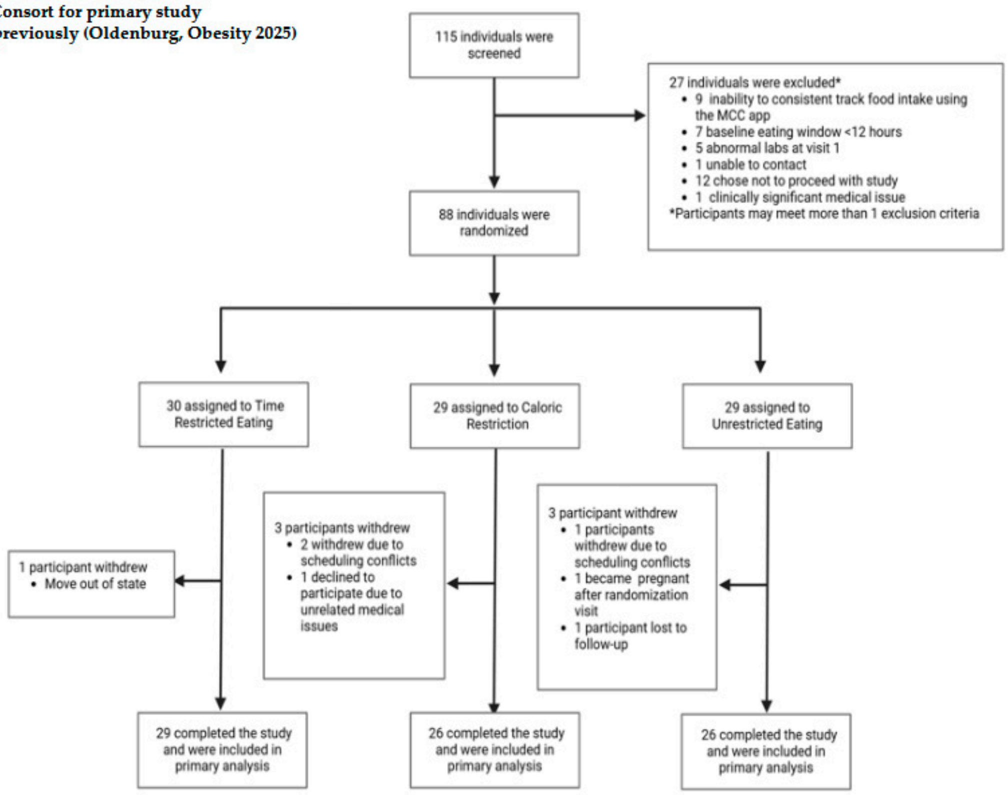

The flow diagram for the primary study was published previously (Oldenburg, Obesity 2025) and replicated here for reference. For this secondary analysis, participants also required ≥7 days of mCC documentation showing ≥2 daily eating events separated by ≥5 hours, recorded >1 day/week, with a ≥12-hour eating window (95% of events), yielding 44 eligible participants (TRE: n=19; CR: n=8; UE: n=17).

Figure S1: Consort for primary study published previously[15].
